# Heritability of cerebellar subregion volumes in adolescent and young adult twins

**DOI:** 10.1002/hbm.26717

**Published:** 2024-05-26

**Authors:** Lachlan T. Strike, Rebecca Kerestes, Katie L. McMahon, Greig I. de Zubicaray, Ian H. Harding, Sarah E. Medland

**Affiliations:** ^1^ Psychiatric Genetics, QIMR Berghofer Medical Research Institute Brisbane Australia; ^2^ School of Psychology and Counselling, Faculty of Health Queensland University of Technology Kelvin Grove Queensland Australia; ^3^ School of Biomedical Sciences, Faculty of Medicine University of Queensland Brisbane Australia; ^4^ Department of Neuroscience, Central Clinical School Monash University Melbourne Australia; ^5^ School of Clinical Sciences, Centre for Biomedical Technologies Queensland University of Technology Brisbane Queensland Australia; ^6^ Cerebellum and Neurodegeneration, QIMR Berghofer Medical Research Institute Brisbane Australia; ^7^ School of Psychology University of Queensland Brisbane Australia

**Keywords:** cerebellum, genetics, heritability, magnetic resonance imaging, twin study

## Abstract

Twin studies have found gross cerebellar volume to be highly heritable. However, whether fine‐grained regional volumes within the cerebellum are similarly heritable is still being determined. Anatomical MRI scans from two independent datasets (QTIM: Queensland Twin IMaging, *N =* 798, mean age 22.1 years; QTAB: Queensland Twin Adolescent Brain, *N =* 396, mean age 11.3 years) were combined with an optimised and automated cerebellum parcellation algorithm to segment and measure 28 cerebellar regions. We show that the heritability of regional volumetric measures varies widely across the cerebellum ($h^{2}$ 47%–91%). Additionally, the good to excellent test–retest reliability for a subsample of QTIM participants suggests that non‐genetic variance in cerebellar volumes is due primarily to unique environmental influences rather than measurement error. We also show a consistent pattern of strong associations between the volumes of homologous left and right hemisphere regions. Associations were predominantly driven by genetic effects shared between lobules, with only sparse contributions from environmental effects. These findings are consistent with similar studies of the cerebrum and provide a first approximation of the upper bound of heritability detectable by genome‐wide association studies.

## Graphical abstract

This research reveals a wide variability in the heritability of specific cerebellar regions (47%–91%). The findings highlight the genetic underpinnings of cerebellar structure and emphasise the substantial influence of unique environmental factors on regional volumetric measures.

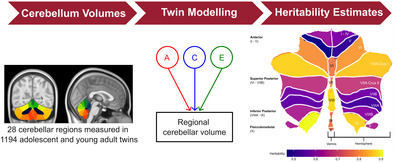

## 1 INTRODUCTION

The cerebellum is crucial in motor control, coordination, and balance (Morton & Bastian, 2004; Stoodley & Schmahmann, 2018; Timmann et al., 2010). There is also clear evidence for cerebellar involvement in affect, language, and social functions (Buckner, 2013; King et al., 2019; Schmahmann, 2019). Cerebellar changes are widely reported in psychiatric conditions (Lupo et al., 2019; Moberget et al., 2018; Zhang et al., 2020) with stronger evidence for a role in neurodevelopmental conditions (Bruchhage et al., 2018; Sathyanesan et al., 2019; Stoodley, 2016). In children, cerebellar morphology has also been associated with general cognitive function and general psychopathology (Moberget et al., 2019). Hughes et al. (2023) recently reported associations between lower grey matter cerebellar volumes and psychiatric symptoms. Interestingly, the same study found that genes associated with a cross‐disorder neurodevelopmental polygenic score were preferentially expressed in the cerebellum.

Twin studies indicate that substantial genetic effects underlie structural variation in the cerebellum. Heritability estimates (*h* ^2^; the proportion of phenotypic variance attributable to genetic variation) for gross cerebellar volumes range from 49% to 88% in children (Maes et al., 2023; Peper et al., 2009; Wallace et al., 2006) and 64% to 85% in adults (Batouli et al., 2014; Kremen et al., 2010; Lukies et al., 2017; Posthuma et al., 2000). Like the cerebrum, the cerebellum can be parcellated into individual structures based on neuroanatomical features. Broadly, the cerebellum can be divided into two hemispheres, with a midline vermis connecting the hemispheres. The hemispheres and vermis can then be divided into lobes (anterior, superior‐posterior, inferior posterior, flocculonodular), which can then, in turn, be subdivided into lobules (numbered using Roman numerals I through X) (Haines & Mihailoff, 2018; Larsell, 1947). In contrast to studies of the cerebrum, studies investigating genetic influences on individual cerebellum regions are sparse.

Chambers et al. (2022) reported moderate single nucleotide polymorphism (SNP)‐based heritability estimates for lobar volumes across the cerebellum in the UK Biobank ($h_{\mathrm{SNP}}^{2}$ 35%–57%). Further, the authors reported moderate shared genetic influence between lobar volumes. However, individual lobule heritability and genetic covariance were not explored, potentially due to questions regarding the accuracy of the lobule volume estimation in the UK Biobank (Chambers et al., 2022; Diedrichsen, 2006). Moreover, SNP‐based heritability estimates explain only the proportion of phenotypic variance explained by common genetic variants, generally resulting in lower heritability estimates (as compared to twin and family‐based studies (Manolio et al., 2009)). Liu et al. (2022) used the Developing Human Connectome Project to estimate twin‐based heritability for T1w/T2w ratio values for individual lobule volumes. The authors reported higher heritability in posterior rather than anterior cerebellar lobules, speculating that this may result from associations between cognitive processing and posterior cerebellum microstructure. It is unknown whether a similar gradient of genetic influences would be expected for volume across cerebellar regions.

Here, we apply an automated cerebellum parcellation algorithm based on convolutional neural networks to two independent and genetically informative imaging datasets. For the first time, we estimate the influence of genetic and environmental factors on variation in the size of individual cerebellum lobules. We focus on volumetric measures of individual regions to elucidate cerebellar morphology in greater detail than previous work using lobar volumes (Chambers et al., 2022) and to complement similar research on tissue microstructure (Liu et al., 2022). As individual lobules may vary in their measurement reliability (potentially influencing estimates of genetic and environmental variance (Ge et al., 2017)), we estimate the test–retest reliability of our imaging measures in a subsample of participants. We then examine associations between cerebellar region volumes and assess the strength of genetic and environmental contributions to these associations.

Based on the heterogenous patterning of genetic influences on the cerebrum (Kremen et al., 2010; Strike, Hansell, Thompson, et al., 2019), we hypothesise that cerebellar regions will differ in their levels of genetic influence. Moreover, we expect these differences will not strongly reflect differences in measurement reliability but in cerebellar function. Specifically, we anticipate higher heritability estimates for regions associated with cognitive processing (i.e., posterior lobules), consistent with past findings for T1w/T2w ratio values (Liu et al., 2022). Like previous results for cortical and subcortical structures, we expect associations between cerebellar regions to be the strongest for homologous left and right hemisphere regions (Eyler et al., 2011; Schmitt et al., 2008; Strike, Hansell, Couvy‐Duchesne, et al., 2019). Further, despite structural and functional asymmetries within the cerebellum (Hu et al., 2008; Saltoun et al., 2023), we hypothesise that overlapping genetic influences will drive these associations, with little evidence of lateralised genetic influence.

## 2 MATERIALS AND METHODS

**FIGURE 1:**
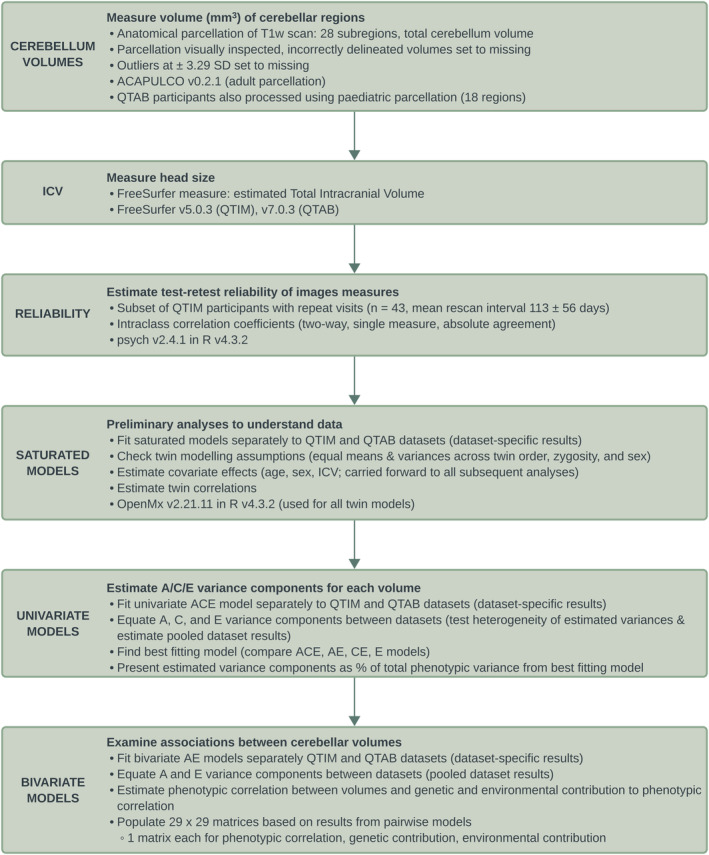
Schematic representation of the imaging and statistical analysis workflow.

Figure 1 summarises the present study's image processing and statistical analysis workflow. The analysis code is available online at https://github.com/PsychiatricGenetics/Cerebellar_heritability.

### 2.1 Participants

Participants were from two independent and genetically informative studies—the Queensland Twin Imaging (QTIM) study of brain structure and function (de Zubicaray et al., 2008) and the Queensland Twin Adolescent Brain (QTAB) longitudinal brain development study (Strike et al., 2023). The pooled dataset comprised 1194 individuals (57% female) from 691 families, including 467 complete twin pairs (208 monozygotic [MZ] and 259 dizygotic [DZ]). Participants ranged in age from 9 to 30 years, with a mean age of 18.5 ± 6.2 years. Exclusion criteria for both studies included neurological and psychiatric conditions, a history of severe head injury, and general MRI contraindications (e.g., metal implants). The QTIM and QTAB studies were research projects and did not include neuroradiological examinations.

#### 2.1.1 QTIM

The QTIM study (Blokland et al., 2017; Couvy‐Duchesne et al., 2016; Hansell, Strike, van Eijk, et al., 2022) collected structural and functional MRI scans in young adults (18–30 years) and a subsample of adolescents (12–16 years). A range of behavioural measures, including cognitive function, exist for most QTIM participants due to their earlier participation in the Brisbane Adolescent Twin Study (aka Brisbane Longitudinal Twin Study) (Wright & Martin, 2004). The QTIM dataset contains imaging data for 682 families (1202 individuals). For the present study, we excluded a subset of participants due to a pervasive imaging artifact at the level of the subtentorial structures (including the cerebellum), resulting in severe image distortion. The final QTIM sample comprised 798 individuals (61% female, mean age 22.1 ± 4.3 years, age range 12–30 years, all right‐handed), including 271 twin pairs (114 monozygotic (MZ) and 157 dizygotic (DZ)), 10 pairs from triplet sets (a single pair of two of the three triplets selected for analysis, creating an additional 1 MZ and 9 DZ pairs), 200 unpaired twins (i.e., participants whose co‐twin either was not scanned or whose data was not useable, but were retained to improve estimated means and variances), and 36 singleton siblings of twins. Genotypic data determined zygosity in same‐sex twins. The study was approved by the Human Research Ethics Committees at the University of Queensland, QIMR Berghofer Medical Research Institute, and UnitingCare Health. Written informed consent was obtained from all participants, including a parent or guardian for those aged under 18 years.

#### 2.1.2 QTAB

The QTAB study (Hansell, Strike, de Zubicaray, et al., 2022; O'Callaghan et al., 2021; Strike et al., 2023) focused on the period of late childhood/early adolescence, with brain imaging, cognition, mental health, and early life/family demographics data collected over two waves (wave 1: 9–14 years, wave 2: 10–16 years). The baseline QTAB wave includes imaging data for 211 families (422 individuals). After excluding participants with poor anatomical scans, the final QTAB sample comprised 396 individuals (51% female, mean age 11.3 ± 1.4 years, age range 9–14 years, 82% right‐handed), including 181 twin pairs (93 MZ and 88 DZ), five pairs from triplet sets (all DZ), and 24 unpaired twins. Zygosity in same‐sex twins was determined by genotypic data (93% of twin pairs) or parental questionnaire (7% of twin pairs). The study was approved by the Human Research Ethics Committees at The University of Queensland and Children's Health Queensland. Written consent was obtained from all participants and a parent/guardian.

### 2.2 Image acquisition

#### 2.2.1 QTIM

T1‐weighted (T1w) 3D whole‐brain images (T1/TR/TE = 700/1500/3.35 ms; FA = 8°, voxel size = 0.94 × 0.94 × 0.90 mm) were acquired for each participant on a 4 T Bruker Medspec whole‐body MRI system paired with a transverse electromagnetic head coil.

#### 2.2.2 QTAB

T1‐weighted 3D whole‐brain images (MP2RAGE sequence, TR/TE = 4000/2.99 ms, TI1/TI2 = 700/2220 ms, FA1/FA2 = 6°/7°, slices = 192, voxel size 0.8 mm isotropic) were acquired for each participant on a 3 T Siemens Magnetom Prisma paired with a 64‐channel head coil. Before cerebellar parcellation, the amplified background noise in the QTAB T1w MP2RAGE images was removed using AFNI (Cox, 1996; Kashyap, 2021).

### 2.3 Cerebellar volumes

We extracted cerebellar volumes for 28 subregions and total cerebellar volume using a standardised pipeline for examining cerebellum grey matter morphometry (a detailed pipeline description is found in Kerestes et al. (2022)). The pipeline uses ACAPULCO v0.2.1 (Han, Carass, et al., 2020) to parcellate the cerebellum and FreeSurfer (Fischl, 2012) to estimate total intracranial volume (ICV; QTIM ICV measures from FreeSurfer v5.0.3, QTAB measures from v7.0.3). Through ACAPULCO, the cerebellum is parcellated, and volumetric measures (mm^3^) are extracted for 28 regions: bilateral lobules I–III, IV, V, and VI; bilateral Crus I and II; bilateral lobules VIIB, VIIIA, VIIIB, IX, and X; vermis VI, VII, VIII, IX, and X; and the corpus medullare, a measure of the central white matter including the deep cerebellar nuclei. All cerebellar segmentations were visually inspected, and incorrectly delineated regions were set to missing. This was followed by quantitative identification of outlier volumes that were greater or less than 3.29 standard deviations from the group mean of each dataset, with outlier volumes set to missing (see Table S1 for the number of excluded and outlier volumes). As the ACAPULCO algorithm was trained using adult data, we additionally extracted volumes in the QTAB dataset using the ACAPULCO paediatric parcellation protocol (Han, Carass, et al., 2020), in which the cerebellum is parcellated into 18 regions.

### 2.4 Reliability

The test–retest reliability of cerebellar volumes was estimated using intraclass correlation coefficients (ICC; two‐way, single measure, absolute agreement) in a subset of QTIM participants with repeat visits (*n* = 43, mean rescan interval 113 ± 56 days). ICCs were calculated using the package psych v2.4.1 (Revelle, 2024) in R v4.3.2 (R Core Team, 2023).

### 2.5 Saturated models

We used a series of univariate saturated models (estimating all possible parameters) to test twin modelling assumptions, examine covariate effects and estimate MZ and DZ twin correlations (represented as *r*MZ and *r*DZ, respectively) for 28 regional cerebellar volumes (and total cerebellar volume) separately in the QTIM and QTAB datasets. Assumption testing examined mean and variance differences between first and second‐born twins, zygosity groups (i.e., MZ female, MZ male, DZ female, DZ male, DZ opposite‐sex), and between twins and singleton siblings of twins (QTIM dataset only). Covariates included age, sex, and ICV; these were carried forward for all subsequent analyses. Models were fit using the maximum‐likelihood structural equation modelling package OpenMx v2.21.11 (Boker et al., 2023; Neale et al., 2016) in R v4.3.2 (R Core Team, 2023). The significance of assumption tests and covariate effects was assessed through likelihood ratio tests comparing the fit between nested models (e.g., comparing models with and without a sex covariate) (Grasby, Verweij, Mosing, Zietsch, & Medland, 2017).

### 2.6 Univariate models

We used the classical twin study design (Neale & Maes, 2004) to estimate genetic and environmental variance in regional cerebellum volumes. The classical twin design contrasts the observed covariance between MZ twins and DZ twins to partition the variance in a phenotype into three sources: additive genetic (A), common or shared environment (C), and residual effects, including idiosyncratic environmental factors and measurement error (E). Variance can also be partitioned into a fourth source: non‐additive genetic (D, e.g., dominance and epistasis). However, D and C are confounded in a classical twin study and require data from additional family members to estimate these effects simultaneously. The present study did not have sufficient power to discriminate between A and D effects (Keller et al., 2010), so models containing D effects were not considered. Consequently, our estimates of A include additive and dominant genetic effects.

Univariate ACE models were first fit to the QTIM and QTAB datasets separately, allowing means and estimated variance components to differ. Constrained models in which estimated variance components were equated were fit to test the heterogeneity of variance components between the datasets (using likelihood ratio tests for significance testing). Simplified sub‐models containing AE, CE, and E variance sources were then fit to the data, with likelihood‐ratio tests and Akaike's Information Criteria used to compare models and select a single best‐fitting model for each cerebellar region. Variance component estimates are presented as a percentage of total phenotypic variance with maximum‐likelihood 95% confidence intervals. We used the direct variance parameterisation of the univariate ACE model (Verhulst et al., 2019), which can produce negative variance component estimates (Maes et al., 2023). Univariate analyses were repeated in the QTAB dataset using volumes from the paediatric parcellation of ACAPULCO (18 regions).

### 2.7 Bivariate models

We then used bivariate twin models to estimate phenotypic (*r*_ph_), genetic (*r*_A_), and unique environmental (*r*_E_) correlations between cerebellar volumes (based on univariate results, common environment effects were not modelled). We fit bivariate models separately to the QTIM and QTAB datasets, initially allowing means and estimated variance components to differ between the datasets (i.e., dataset‐specific results) before equating the estimated variance components between the datasets (i.e., pooled dataset results). Genetic and environmental correlations reflect the degree of shared or overlapping genetic and environmental variance between two phenotypes. However, it is essential to note that the contribution of genetic (or environmental) covariance to phenotypic associations is relative to the proportion of phenotypic variance explained by genetic (or environmental) effects. For instance, while a high genetic correlation may be observed between two phenotypes, if neither phenotype is sufficiently heritable, the overlapping genetic variance contributes little by way of a phenotypic association (i.e., the high genetic correlation is misleading). As a solution, we examined shared genetic influence between cerebellar volumes by calculating the genetic contribution to the phenotypic correlation (*r*_ph‐a_):

$$\sqrt{h_{1}^{2}}\times r_{A}\times \sqrt{h_{2}^{2}}$$

Here, $h_{1}^{2}$ and $h_{2}^{2}$ represent the heritability of phenotype 1 and 2, respectively, and $r_{A}$ the genetic correlation between phenotype 1 and 2. We similarly calculated the environmental contribution to the phenotypic correlation (*r*_ph‐e_). The significance of the associations was tested by setting the covariance of interest (i.e., phenotypic, genetic, environmental) to zero and testing whether this significantly affected model fit (assessed via likelihood ratio tests). Phenotypic correlations and the genetic and environmental contributions to the phenotypic correlations were estimated for all possible pairs of cerebellar volume measures (28 cerebellar regions plus total cerebellum volume). The results of these pairwise models were used to populate a series of 29 by 29 correlation matrices (one each for phenotypic correlations, genetic contribution to the phenotypic correlation, and environmental contribution to the phenotypic correlation).

### 2.8 Multiple testing correction

We used the Benjamini–Hochberg procedure to control the false discovery rate for the multiple comparisons (Benjamini & Hochberg, 1995). The procedure was applied separately for (1) covariate effects (i.e., age, sex, ICV), (2) assumption testing (i.e., means and variance differences), (3) fitting of univariate models (i.e., equating estimated A/C/E variance components and fitting AE, CE, E sub‐models), and (4) bivariate model covariances (i.e., phenotypic, genetic, environmental).

## 3 RESULTS

### 3.1 Preliminary analyses

Means, standard deviations, and covariate effects for the 28 cerebellar volumes are presented in Table S1. Both datasets showed sparse and small effects of age on cerebellar volumes. When controlling for ICV, larger volumes were found in males than females for 16/28 regions in the QTIM dataset and 5/28 regions in the QTAB dataset (left Crus I and vermis III showed consistent sex effects in both datasets). ICV was positively associated with cerebellar volumes in both datasets. Assumption testing showed volumes were similar across twins/siblings and zygosity groups for all cerebellar regions. MZ twin correlations were larger than corresponding DZ twin correlations for all regions in both datasets (Tables S2 and S3), suggesting a genetic influence on variation in all cerebellar volumes. Test–retest reliability estimates in the QTIM dataset ranged from good to excellent (0.77–0.98; Table 1 and Figure 3b).

**TABLE 1:** AE model variance component estimates (standardised to total phenotypic variance, with 95% confidence intervals) in the pooled dataset and test–retest reliability estimates in the QTIM dataset.

	Variance estimates from AE model (95% CI)		QTIM reliability (95% CI)
	A	E	
Anterior			
Left I–III	.64 (.56, .71)	.36 (.29, .44)	.96 (.93, .98)
Right I–III	.60 (.51, .68)	.40 (.32, .49)	.92 (.85, .95)
Left IV	.61 (.53, .68)	.39 (.32, .47)	.92 (.86, .96)
Right IV	.57 (.48, .65)	.43 (.35, .52)	.84 (.72, .91)
Left V	.47 (.36, .56)	.53 (.44, .64)	.87 (.77, .92)
Right V	.50 (.41, .58)	.50 (.42, .59)	.91 (.84, .95)
Superior posterior			
Left VI	.80 (.75, .84)	.20 (.16, .25)	.96 (.93, .98)
Right VI	.81 (.77, .85)	.19 (.15, .23)	.96 (.93, .98)
Left crus I	.87 (.83, .89)	.13 (.11, .17)	.98 (.97, .99)
Right crus I	.86 (.82, .89)	.14 (.11, .18)	.95 (.90, .97)
Left crus II	.67 (.59, .74)	.33 (.26, .41)	.91 (.85, .95)
Right crus II	.68 (.61, .75)	.32 (.25, .39)	.92 (.85, .95)
Left VIIB	.57 (.47, .65)	.43 (.35, .53)	.91 (.83, .95)
Right VIIB	.57 (.48, .64)	.43 (.36, .52)	.84 (.73, .91)
Inferior posterior			
Left VIIIA	.55 (.46, .63)	.45 (.37, .54)	.88 (.79, .93)
Right VIIIA	.52 (.43, .60)	.48 (.40, .57)	.77 (.61, .87)
Left VIIIB	.64 (.56, .70)	.36 (.30, .44)	.91 (.84, .95)
Right VIIIB	.60 (.52, .67)	.40 (.33, .48)	.91 (.84, .95)
Left IX	.91 (.88, .92)	.09 (.08, .12)	.96 (.92, .98)
Right IX	.87 (.84, .90)	.13 (.10, .16)	.95 (.92, .97)
Flocculonodular			
Left X	.64 (.56, .71)	.36 (.29, .44)	.93 (.87, .96)
Right X	.64 (.56, .71)	.36 (.29, .44)	.84 (.73, .91)
Vermis			
Vermis VI	.77 (.71, .81)	.23 (.19, .29)	.94 (.89, .97)
Vermis VII	.77 (.72, .81)	.23 (.19, .28)	.96 (.92, .98)
Vermis VIII	.88 (.85, .90)	.12 (.10, .15)	.95 (.90, .97)
Vermis IX	.76 (.70, .80)	.24 (.20, .30)	.93 (.88, .96)
Vermis X	.74 (.69, .79)	.26 (.21, .31)	.95 (.91, .97)
White matter			
Corpus medullare	.89 (.86, .91)	.11 (.09, .14)	.92 (.85, .96)
Global			
Total cerebellar	.91 (.89, .93)	.09 (.07, .11)	.95 (.92, .97)

*Note*: Twin models included corrections for effects of age, sex, and ICV, with separate means for QTIM and QTAB participants but equated estimated variance components.

### 3.2 Variance component estimates

Variance component estimates for cerebellar volumes were obtained from univariate twin models correcting for age, sex, and ICV. The data from the QTIM and QTAB datasets were modelled separately, allowing means and estimated variance components to differ between the two datasets. We then fit increasingly constrained models to test the heterogeneity of the estimated variance components between the datasets and find the most parsimonious model. Results showed that equating the A, C, and E variance components across datasets did not significantly reduce model fit, except for right VIIB and total cerebellum volumes. We emphasise that this does not invalidate variance estimates for these regions but rather provides evidence that the magnitude of variance components for these two regions differs between the QTAB and QTIM datasets (dataset‐specific estimated variance components presented in Tables S2 and S3 and Figure S1). Estimates of the general common environment effect (i.e., C equated across the two datasets) were small (≤21%), and model comparisons suggested insufficient power to detect significance despite the point estimates. Hence, models specifying only A and E variance components were selected as the best‐fitting model for all cerebellar volumes. We note that our decision to drop C effects could overestimate A effects for some cerebellar volumes, and we provide the full ACE/ADE models in Table S4.

**FIGURE 2:**
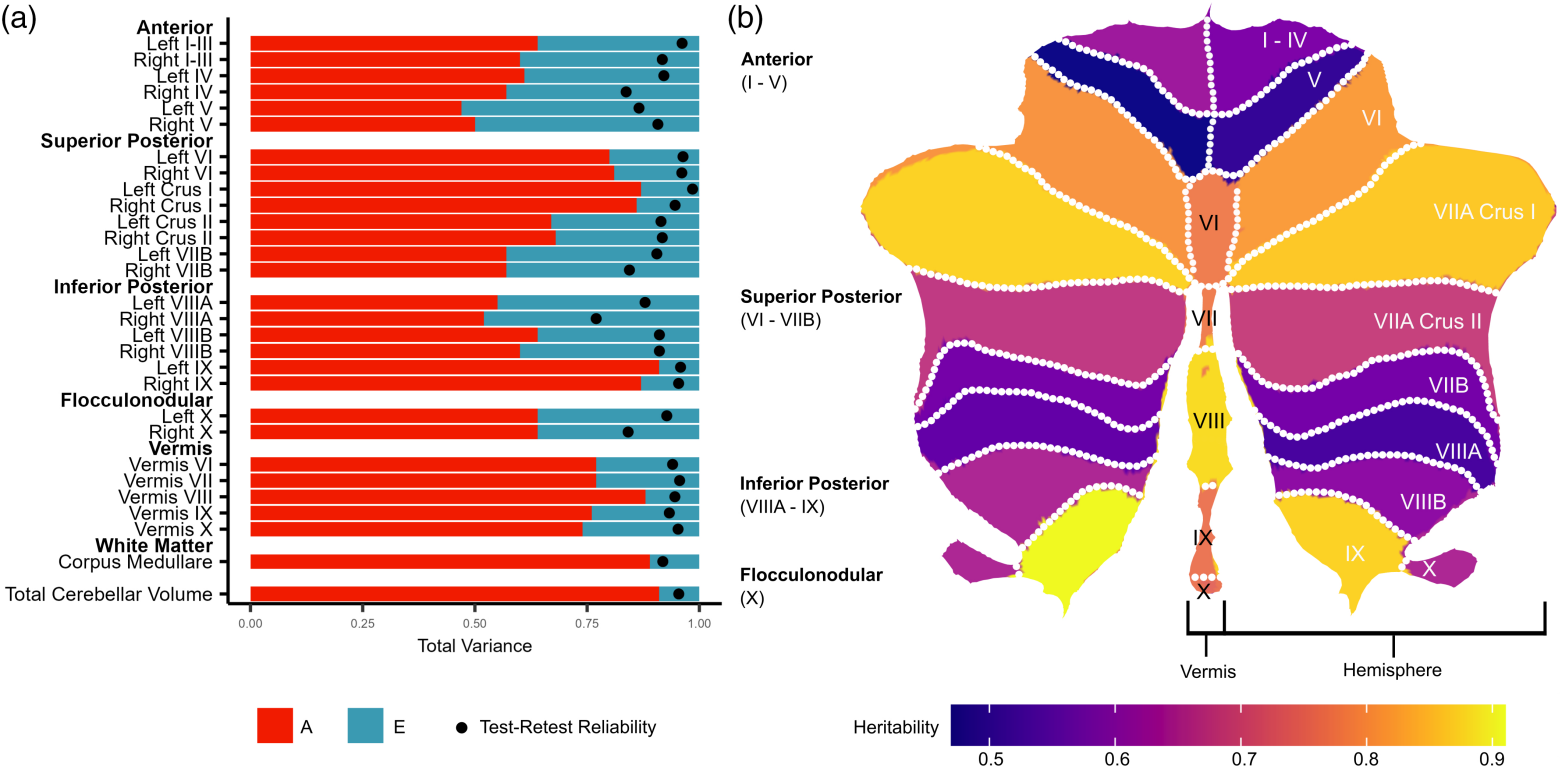
Genetic (a, b) and environmental (a) variance estimates (presented as a proportion of total phenotypic variance) for 28 regional cerebellar volumes (and total cerebellum volume) in the pooled dataset. Twin models included corrections for effects of age, sex, and ICV (with separate means for QTIM and QTAB participants) and specified only additive genetic (A) and unique environmental (E) sources of variance. Black dots (a) represent test–retest reliability estimates in the QTIM dataset (*n* = 43, scans approximately 3 months apart). Panel (b) created using the SUIT toolbox (Diedrichsen & Zotow, 2015); due to the small size of lobules I–III, the mean of lobules I–III and IV is displayed in (b) (i.e., lobule I–IV).

Heritability estimates ranged from 47% (left lobule V) to 91% (left lobule IX; Figure 2 and Table 1), and estimates were similar across homologous left and right hemisphere regions. For 21 out of 28 regions, estimates of genetic effects were greater (i.e., 95% confidence intervals did not overlap) than corresponding unique environmental effects (which includes measurement error). Interestingly, there were substantial differences in heritability estimates within lobes, particularly for the superior and inferior posterior lobes. Furthermore, there was a different pattern of heritability estimates across the three subregions of lobule VII (i.e., Crus I, Crus II, VIIB). Notably, regions with moderate heritability estimates (*h* ^2^ 47%–60%) did not show low QTIM test–retest reliability estimates (ICC 0.77–0.92; Table 1), suggesting that the more moderate heritability estimates for these regions were not a result of large measurement error.

Total cerebellum volume was highly heritable (A = 91%). Heritability estimates for cerebellar volumes extracted using the adult and paediatric parcellations in the QTAB dataset were comparable (Table S5); however, volumes for left and right lobule X were more heritable in the paediatric parcellation. Further, there were fewer incorrectly delineated regions using the paediatric parcellation protocol (which delineates the cerebellum into a smaller number of subregions; Table S5).

### 3.3 Associations between cerebellar volumes

We then used bivariate twin models to estimate phenotypic correlations (*r*_ph_) between cerebellar volumes and the genetic (*r*_ph‐a_) and unique environmental (*r*_ph‐e_) contributions to these correlations (based on univariate results, common environment effects were modelled). We first fit bivariate models separately to the QTIM and QTAB datasets, allowing means and estimated variance components to differ. Results were remarkably similar between datasets, though there were more significant associations in the QTIM dataset (Figures S2 and S3).

**FIGURE 3:**
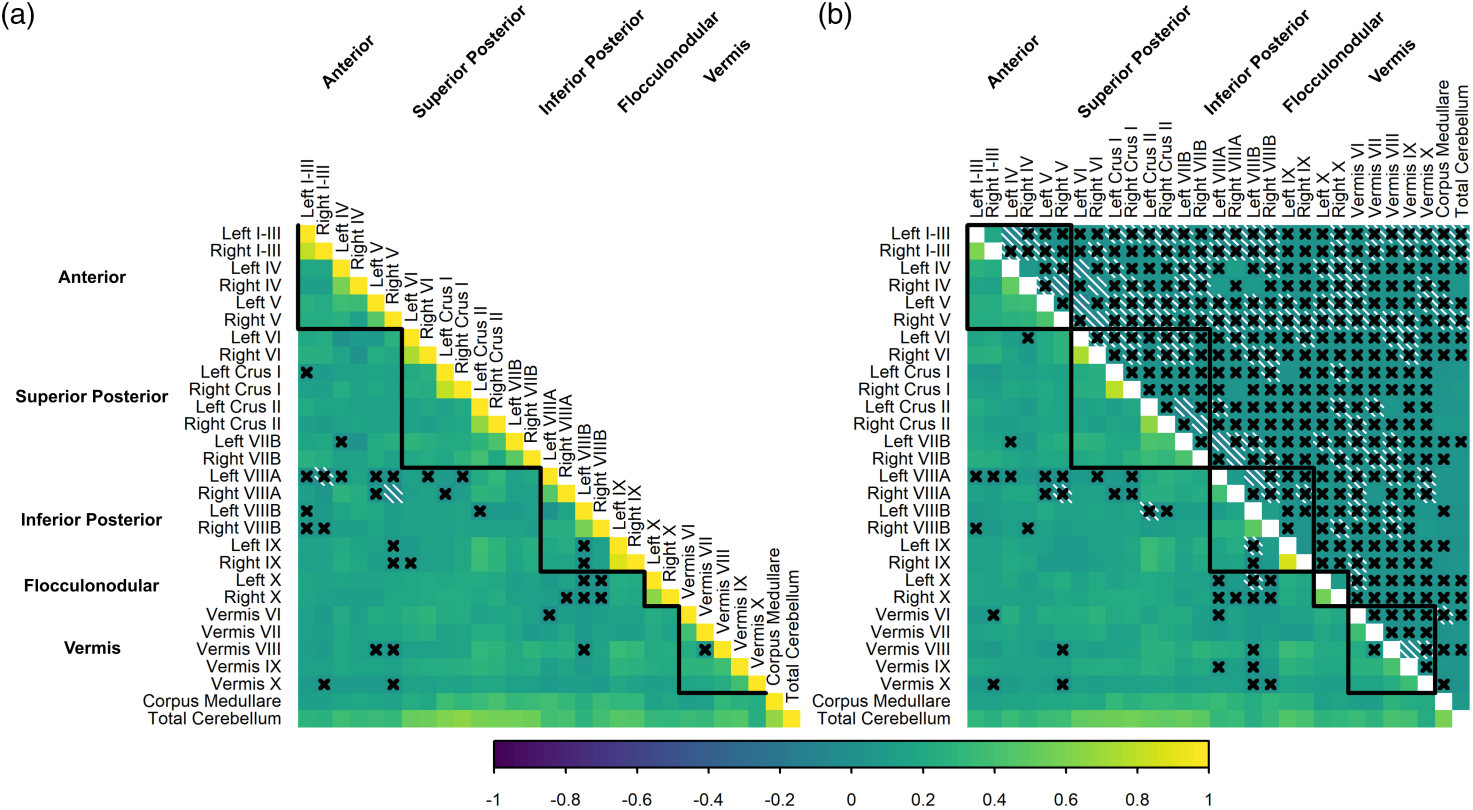
Phenotypic correlations (a), with genetic (lower) and environmental (upper) contributions to the phenotypic correlations (b), for cerebellar volume across 28 regions and total cerebellum volume in the pooled dataset. Lobules are presented in groups (anterior, superior posterior, inferior posterior, flocculonodular, vermis). Non‐significant correlations/contributions are denoted ×; negative correlations/contributions are shaded white). Twin models included corrections for effects of age, sex, and ICV (with separate means for QTIM and QTAB participants, but equated estimated variance components) and specified only additive genetic (A) and unique environmental (E) sources of variance.

Estimated variance components were then equated between the two datasets to examine estimates in the pooled dataset. Phenotypic correlations between corresponding left/right regions ranged from .48 (lobule VIIIA) to .89 (lobule IX), with a strong genetic contribution to these associations (*r*_ph‐a_ range = .39–.86; Figure 3a). Phenotypic correlations between cerebellar regions within the same lobe (*r*_ph_ range = .01–.42) were weaker than homologous left and right hemisphere region correlations and slightly stronger than phenotypic correlations between cerebellar regions from different lobes (*r*_ph_ range = −.07 to .37). All subregion volumes were correlated with total cerebellum volume (*r*_ph_ .27–.67). Genetic contributions largely accounted for phenotypic correlations between cerebellar regions. However, sparse patterns of unique environmental covariance were present, particularly between the volumes of homologous left and right hemisphere regions.

## 4 DISCUSSION

To further our understanding of the mechanisms underlying cerebellar neurogenesis, we estimated genetic and environmental variation in the volume of 28 cerebellar regions in adolescent and young adult twins and singletons. There was a moderate to strong genetic influence for all regional cerebellar volumes, with heritability estimates ranging from 47% to 91%. Most striking were the substantial differences in heritability estimates within lobes, particularly for the superior and inferior posterior lobes. There was little evidence of common (shared) environmental variance. Phenotypic correlations between volumes of homologous left and right hemisphere regions were moderate to strong, driven predominantly by shared genetic influences.

The heritability estimate for total cerebellum volume was 91%, in line with past twin‐based estimates (Peper et al., 2009; Posthuma et al., 2000). Similarly, the estimate for corpus medullare volume (*h* ^2^ 89%) was consistent with past estimates for cerebellar white matter volume (Kremen et al., 2010). Heritability estimates for regional cerebellar volumes were similar to estimates for regional cortical (Kremen et al., 2010; Strike, Hansell, Thompson, et al., 2019) and subcortical volumes (den Braber et al., 2013; Renteria et al., 2014). Further, heritability estimates in the present study were higher than estimates for regional T1w/T2w ratio values (a proxy measure of myelin content (Liu et al., 2022)) and lobar volumes (n.b. SNP‐based heritability (Chambers et al., 2022)). We also found little evidence of shared environmental effects, consistent with past studies of volumetric brain measures (Kremen et al., 2010; Schmitt et al., 2008; Strike, Hansell, Thompson, et al., 2019).

We found a wide range of heritability estimates across the cerebellum (*h* ^2^ 47%–91%). Lower heritability estimates could reflect weaker genetic influence, stronger environmental influence (including measurement error), or both, as heritability is presented relative to total phenotypic variance. Notably, we showed that regions of lower heritability were unlikely to be the result of large measurement unreliability, with such regions (e.g., bilateral lobule V, bilateral lobule VIIB; *h* ^2^ ≤ 57%) exhibiting good reliability in the QTIM test–retest dataset (ICC ≥ 0.84). However, this is not to say that heritability estimates are wholly unaffected by measurement reliability or that repeatability is the only contributor to measurement error (i.e., test–retest correlations do not measure the accuracy of cerebellar segmentation).

The present study finds higher heritability estimates for lobules VI, Crus I, and IX than for lobules I–III, IV, V, and VIII. Studies have suggested that sensorimotor representation is related to anterior lobules (i.e., I–III, IV, V) and lobule VIII, whereas posterior lobules (i.e., VI, Crus I) and lobule IX are involved in cognitive functions (Guell, Gabrieli, et al., 2018; Stoodley & Schmahmann, 2018). More substantial genetic influence over cerebellar regions associated with cognitive function is plausible; indeed, cognitive abilities are substantially influenced by genes (Davies et al., 2011; Haworth et al., 2010; Mollon et al., 2021), and there is an established coupling between posterior cerebellar lobules and prefrontal/parietal cortices (Bernard et al., 2012; Kelly & Strick, 2003; Salmi et al., 2010). Further, cerebellar structural plasticity induced by motor skill learning (Hutchinson et al., 2003; Woodruff‐Pak et al., 2001) could underlie anterior lobe volumes' lower heritability (conversely, stronger environmental influence). However, lobule VIIB, which has shown associations with visual working memory (Brissenden et al., 2021) and executive function (Stoodley & Schmahmann, 2009), was among the regions of lowest heritability, suggesting that an opposite viewpoint (e.g., higher environmental variance due to plasticity) is equally plausible.

Macroscale structure–function relationships in the cerebellum are also characterised through functional gradient approaches. Using resting‐state fMRI data from the Human Connectome Project (HCP), Guell, Schmahmann, et al. (2018) demonstrated a functional gradient with cerebellar motor regions at one end (lobules IV, V, VI, VIII) and regions associated with the HCP language task at the other end (posterior Crus I and Crus II, IX). We observe some similarity between this functional gradient and the heritability estimates of the present study; heritability estimates are lower for lobules IV, V, and VIII and higher for lobules Crus I and IX. While comparing functional gradients with lobule‐based heritability estimates is difficult (as functional gradients are freely estimated and not constrained to anatomical boundaries), these similarities warrant further investigation in larger samples through voxel‐wise heritability estimates of structural and functional cerebellar phenotypes.

Differences in heritability estimates may also reflect developmental differences between cerebellar subregions. Gaiser et al. (2024) recently published cerebellar growth models in 4862 children and adolescents (6–17 years). Here, the authors showed smaller age‐related effects on cerebellar volume for anterior lobules (III–V), and larger age‐related effects in posterior lobules (VI–X) and the corpus medullare. In a smaller sample (50 participants aged 5–24 years), Tiemeier et al. (2010) showed volume trajectories peaked last for the superior posterior lobe (VI, Crus I) and corpus medullare. Interestingly, we found lower heritability estimates for anterior lobules and higher heritability estimates for some posterior lobules (VI, Crus I, IX) and the corpus medullare. Lower heritability estimates for earlier developing brain regions could reflect sustained environmental influences following maturation, with later developing regions experiencing stronger relative genetic effects as they continue to develop. Regions showing large age‐related effects in Gaiser et al. (2024) (e.g., lobule X) but smaller heritability estimates in the present study require further investigation. Interestingly, there was not a clear difference in heritability estimates between phylogenetically older (i.e., flocculonodular and vermis) and newer (i.e., anterior, inferior and superior posterior) regions; however, it must be noted that the cerebellar regions of the present study do not fully represent phylogenetic divisions (i.e., vestibulocerebellum, spinocerebellum, and cerebrocerebellum).

Our finding of a substantial genetic correlation between corresponding left/right cerebellar regions is consistent with previous cortical (Schmitt et al., 2008; Strike, Hansell, Couvy‐Duchesne, et al., 2019; Wen et al., 2016) and subcortical measures (Eyler et al., 2011; Renteria et al., 2014). This strong interhemispheric relationship suggests that genetic influences on cerebellar volumes in corresponding hemispheres are not lateralised (at the macro level). The pattern of relatively homogenous phenotypic correlations across non‐homologous cerebellar regions and the substantial genetic contribution to these associations likely reflect the presence of a global genetic factor influencing variation across the cerebellum. Indeed, controlling for total cerebellum volume (TCV) in place of ICV reveals a more complex pattern of associations across the cerebellum (Figure S4). Here, the strong associations between the volumes of homologous lobules remain. In contrast, associations between other inter‐ and intra‐hemispheric regions are reduced (over 60% of these associations are now non‐significant), and roughly two‐thirds are negative. Importantly, shared genetic influences remain the predominant driver of these associations.

Sex differences (controlling for ICV) were more prominent in the QTIM dataset (age range 12–30 years) than in the QTAB dataset (9–14 years); however, all significant sex effects were in the same direction (i.e., larger for males than females). This result is consistent with Tiemeier et al. (2010), who reported larger inferior and superior posterior lobe volumes in males than in females (ages 5–24 years, controlling for total cerebral volume). Within a similar age range (8–30 years), Koolschijn and Crone (2013) reported larger cerebellar grey matter volume in males than in females (controlling for ICV). The sparse sex effects in the QTAB dataset are similar to the results in adolescents reported by Isiklar et al. (2023) and Gaiser et al. (2024). However, these results are inconsistent with the findings of Rice et al. (2023), in which a greater number of regions showed significant sex effects (controlling for ICV) in similarly aged participants. This discrepancy may result from the greater ratio of male to female participants and the paediatric‐specific cerebellar atlas used by Rice et al. (2023). Interestingly, sex effects are less widespread in lifespan (Romero et al., 2021) and older adult (Han, An, et al., 2020) studies, suggesting that the sex effects of the present study may reflect differences in brain maturation between males and females, particularly pubertal effects (Tiemeier et al., 2010; Wang et al., 2023).

Sex effects due to differences in developmental trajectories may also explain why sex effects were less prominent in the QTAB dataset. Indeed, the age at which peak volume for the total cerebellum volume is reached, reported by Tiemeier et al. (2010) (11.8 and 15.5 years for females and males, respectively), falls outside the mean age of QTAB participants (11.3 years). We found only minor effects of age on cerebellar volumes, comparable to results in a similar paediatric sample (Rice et al., 2023) and consistent with findings showing cerebellar age effects are more prominent in middle to older age (Bernard & Seidler, 2014). Further, we were limited to examining cross‐sectional age effects; longitudinal growth trajectories are better placed to track cerebellar development during adolescence (Gaiser et al., 2024).

Several other automated cerebellar segmentation pipelines exist, including CERES (Romero et al., 2017) and CerebNet (Faber et al., 2022). The ACAPULCO version used in the present (v0.2.1) study has shown lower repeatability and replicability compared to CERES (Soros et al., 2021). This increased variability likely arises from a stochastic algorithm used in ACAPULCO v0.2.1 during MNI registration. However, comparing test–retest reliability estimates in the QTIM dataset using ACAPULCO versions 0.2.1 and 0.3.0 (a patched version) shows very similar estimates between the two versions (Table S6). This finding suggests that while the randomness present in ACAPULCO v0.2.1 may introduce some variability in repeat analyses of the same scan (i.e., repeatability) or analyses of a same‐day repeat scan (i.e., replicability), it is unlikely to reduce our ability to measure similarity between twin pairs (as twin similarity is unlikely to be as high as the similarity between the same participant scanned twice). Further, CerebNet recently showed statistically significant improvements compared with ACAPULCO (Faber et al., 2022). However, we emphasise that in this comparison ACAPULCO exhibited high accuracy and reliability (Faber et al., 2022), demonstrating that it remains a leading cerebellum parcellation pipeline. Interestingly, a novel multimodal (i.e., T1w + T2w) cerebellar segmentation pipeline has shown promising results, potentially further improving cerebellum lobule segmentation (Morell‐Ortega et al., 2024). The recent proliferation of cerebellum segmentation pipelines is a welcomed development for neuroimaging genetics, and future studies are required to examine whether heritability estimates are consistent across these different segmentation approaches.

The cerebellar parcellation used in the present study is based on anatomical divisions, which do not entirely represent cytoarchitectural and functional variation within the cerebellum (Cerminara et al., 2015; Guell, 2022; King et al., 2019) and do not include cerebellar white matter structures (i.e., cerebellar peduncles; (van Baarsen et al., 2016)). While cerebellar morphology can be examined without the constraint of anatomical boundaries (i.e., through voxel‐wise approaches), we elected to use a lobule approach to maximise statistical power in our small (twin) sample by limiting the number of multiple comparisons. In addition, the higher heritability estimates for lobule X, as measured using the paediatric protocol, suggest that the validity of volume estimation within this region may be reduced when using the adult protocol on younger participants. Finally, multi‐dataset heritability can be estimated through meta‐analysis (Kochunov et al., 2014; Pizzagalli et al., 2020). As our analyses involved only two datasets, we elected to examine heritability by pooling the QTIM and QTAB datasets (which additionally facilitated testing the heterogeneity of estimated variance components). Nonetheless, we find nearly identical heritability estimates based on a meta‐analysis of the separate QTIM and QTAB heritability estimates (Table S7).

## 5 CONCLUSIONS

A complex pattern of genetic and environmental factors influences variation in the volume of cerebellar regions. Associations between cerebellar regions are moderate and driven predominantly by genetic effects, potentially reflecting a global factor influencing cerebellar volumes. The twin‐based estimates presented here are a first approximation of the upper bound of heritability detectable by genome‐wide association studies. Identifying specific genetic variants and biological pathways influencing cerebellar volumes is a challenging next step.

## AUTHOR CONTRIBUTIONS

**Lachlan T. Strike**: conceptualization, methodology, data curation, writing—original draft, visualization. **Rebecca Kerestes**: writing—review & editing. **Katie L. McMahon**: writing—review & editing. **Greig de Zubicaray**: writing—review & editing. Ian H. Harding: writing—review & editing. **Sarah E. Medland**: conceptualization, writing—review & editing.

## CONFLICT OF INTEREST STATEMENT

All of the authors have no conflicts of interest to declare.

## Supporting information


**FIGURE S1:** AE model genetic and environmental variance estimates (presented as a proportion of total phenotypic variance) for 28 regional cerebellar volumes (and total cerebellum volume) in the QTIM and QTAB datasets. Twin models included corrections for effects of age, sex, and ICV.
**FIGURE S2.** Phenotypic correlations (a), with genetic (lower) and environmental (upper) contributions to the phenotypic correlations (b), for cerebellar volume across 28 regions and total cerebellum volume in the QTIM dataset. Lobules are presented in groups (anterior, superior posterior, inferior posterior, flocculonodular, vermis). Non‐significant correlations/contributions are denoted × (significance adjusted for multiple comparisons), and negative estimates are shaded white. Twin models included corrections for effects of age, sex and ICV and specified only additive genetic (A) and unique environmental (E) sources of variance.
**FIGURE S3.** Phenotypic correlations (a), with genetic (lower) and environmental (upper) contributions to the phenotypic correlations (b), for cerebellar volume across 28 regions and total cerebellum volume in the QTAB dataset. Lobules are presented in groups (anterior, superior posterior, inferior posterior, flocculonodular, vermis). Non‐significant correlations/contributions are denoted × (significance adjusted for multiple comparisons), and negative estimates are shaded white. Twin models included corrections for effects of age, sex and ICV and specified only additive genetic (A) and unique environmental (E) sources of variance.
**FIGURE S4.** Phenotypic correlations (a), with genetic (lower) and environmental (upper) contributions to the phenotypic correlations (b), for cerebellar volume across 28 regions in the pooled dataset (separate means, equated variance components). Lobules are presented in groups (anterior, superior posterior, inferior posterior, flocculonodular, vermis). Non‐significant correlations/contributions are denoted ×; negative correlations/contributions are shaded white. Twin models included corrections for effects of age, sex and total cerebellar volume (in place of ICV) and specified only additive genetic (A) and unique environmental (E) sources of variance.

**TABLE S1:** Excluded volumes, volume mean, standard deviation, and covariate effects estimated from saturated models for the QTIM and QTAB datasets.
**TABLE S2.** Twin correlations and variance component estimates (relative to total phenotypic variance) and model fit statistics (AIC, LRT) for the QTIM dataset.
**TABLE S3.** Twin correlations and variance component estimates (relative to total phenotypic variance) and model fit statistics (AIC, LRT) for the QTAB dataset.
**TABLE S4.** Variance component estimates (relative to total phenotypic variance) and model fit statistics (AIC, LRT) for the pooled dataset.
**TABLE S5.** Volume mean, standard deviation, twin correlations, and variance component estimates (relative to total phenotypic variance) for paediatric and adult parcellation volumes in the QTAB dataset.
**TABLE S6.** Test–retest reliability estimates in the QTIM dataset (*n* = 43) using ACAPULCO v0.2.1 and v0.3.0.
**TABLE S7.** Meta‐analysis heritability estimates with 95% confidence intervals.

## Data Availability

MRI data for the QTIM and QTAB projects are freely available from the OpenNeuro data repository (Strike, Blokland, et al., 2022; Strike, Hansell, et al., 2022). A data transfer/usage agreement is required to access restricted participant data (i.e., zygosity).
