# Supplementary material for: Heritability of cerebellar subregion volumes in adolescent and young adult twins
Source: Hum Brain Mapp. 2024 May 26;45(8):e26717. doi: 10.1002/hbm.26717 (PMC11128777; doi:10.1002/hbm.26717)
Supplement: Supplementary file 1 — FIGURE S1: AE model genetic and environmental variance estimates (presented as a proportion of total phenotypic variance) for 28 regional cerebellar volumes (and total cerebellum volume) in the QTIM and QTAB datasets. Twin models included corrections for effects of age, sex, and ICV. FIGURE S2. Phenotypic correlations (a), with genetic (lower) and environmental (upper) contributions to the phenotypic correlations (b), for cerebellar volume across 28 regions and total cerebellum volume in the QTIM dataset. Lobules are presented in groups (anterior, superior posterior, inferior posterior, flocculonodular, vermis). Non‐significant correlations/contributions are denoted × (significance adjusted for multiple comparisons), and negative estimates are shaded white. Twin models included corrections for effects of age, sex and ICV and specified only additive genetic (A) and unique environmental (E) sources of variance. FIGURE S3. Phenotypic correlations (a), with genetic (lower) and environmental (upper) contributions to the phenotypic correlations (b), for cerebellar volume across 28 regions and total cerebellum volume in the QTAB dataset. Lobules are presented in groups (anterior, superior posterior, inferior posterior, flocculonodular, vermis). Non‐significant correlations/contributions are denoted × (significance adjusted for multiple comparisons), and negative estimates are shaded white. Twin models included corrections for effects of age, sex and ICV and specified only additive genetic (A) and unique environmental (E) sources of variance. FIGURE S4. Phenotypic correlations (a), with genetic (lower) and environmental (upper) contributions to the phenotypic correlations (b), for cerebellar volume across 28 regions in the pooled dataset (separate means, equated variance components). Lobules are presented in groups (anterior, superior posterior, inferior posterior, flocculonodular, vermis). Non‐significant correlations/contributions are denoted ×; negative correlations/cont [file HBM-45-e26717-s002.docx]

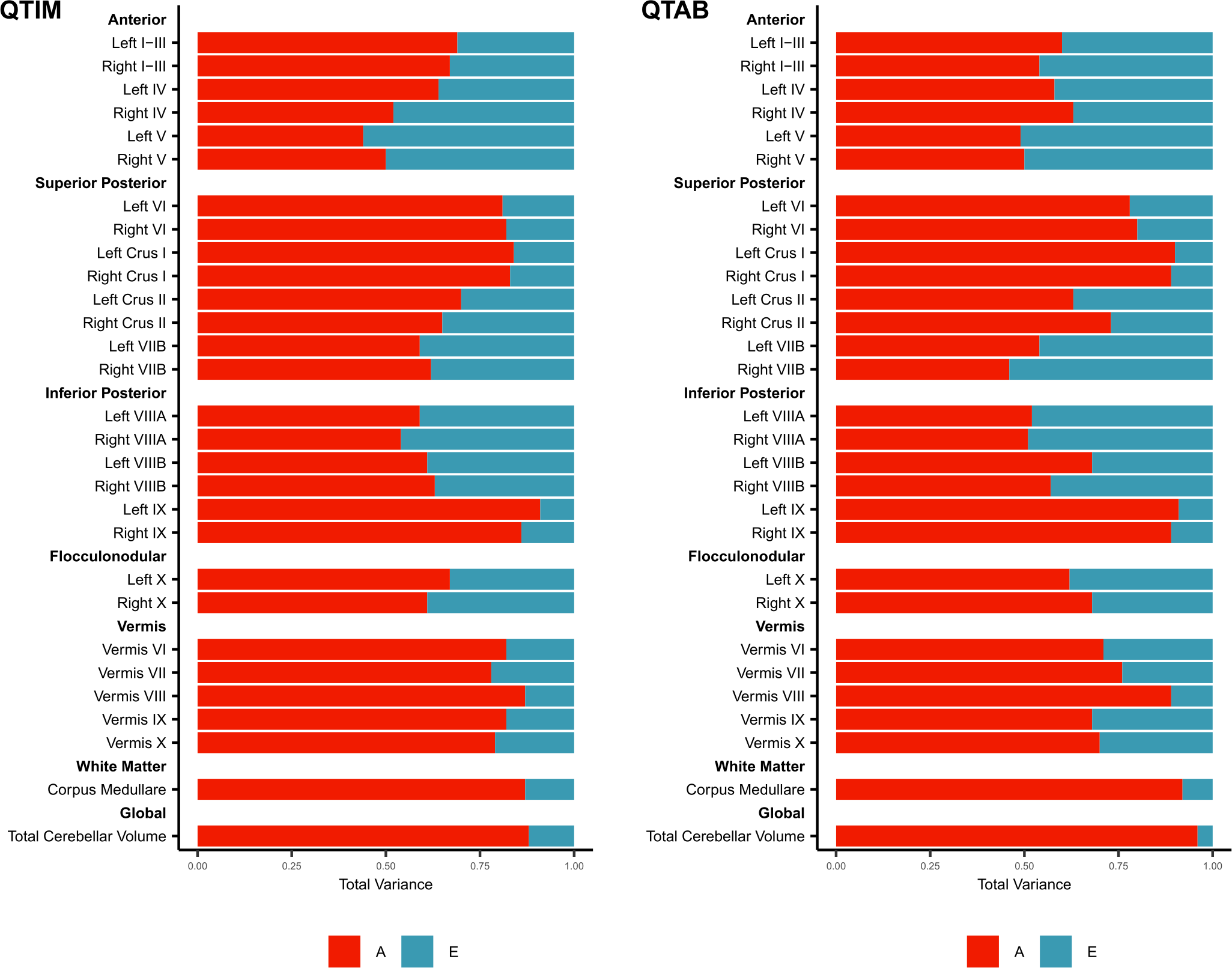


**FIGURE S1** AE model genetic and environmental variance estimates (presented as a proportion of total phenotypic variance) for 28 regional cerebellar volumes (and total cerebellum volume) in the QTIM and QTAB datasets. Twin models included corrections for effects of age, sex, and ICV.


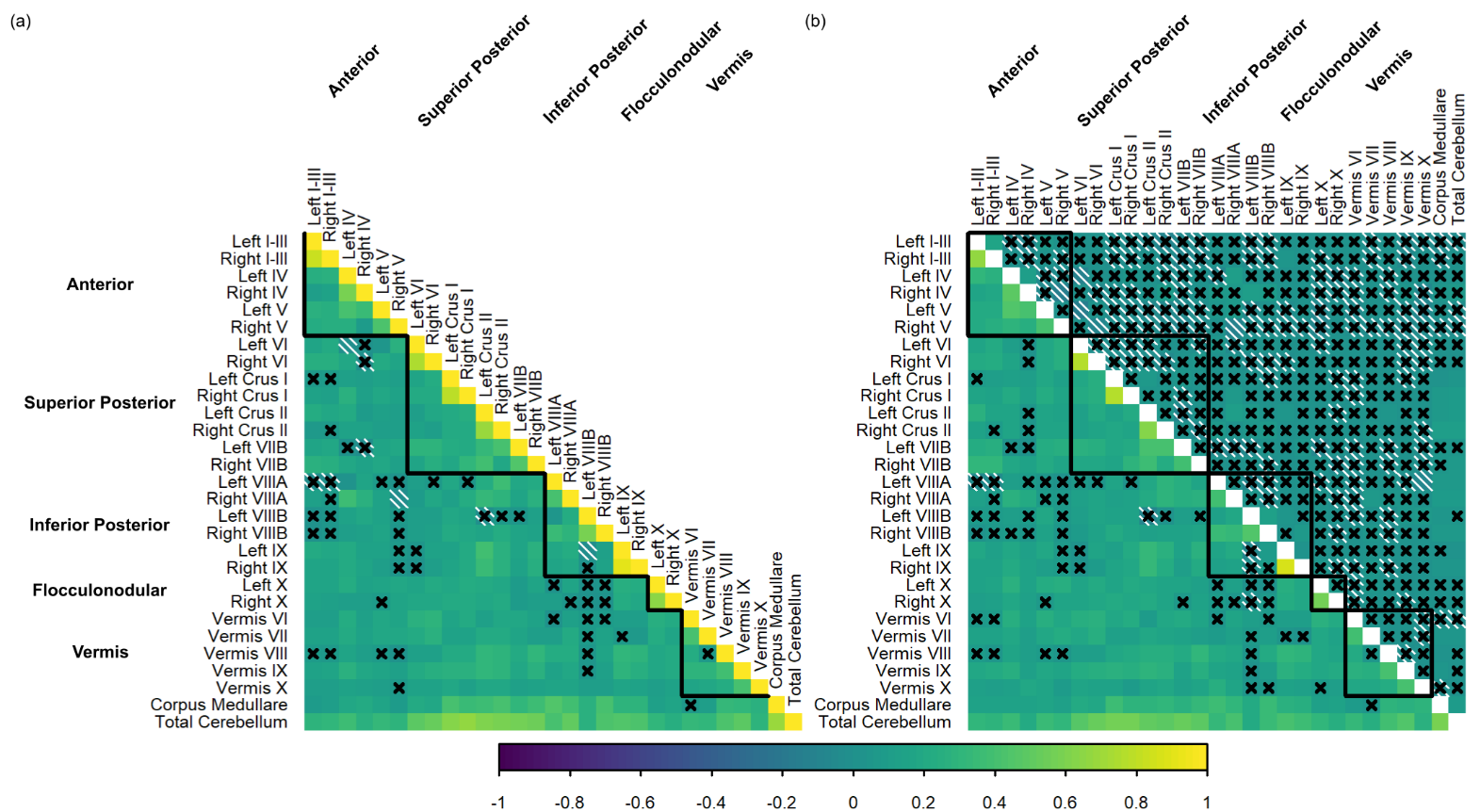


**FIGURE S2** Phenotypic correlations (**a**), with genetic (lower) and environmental (upper) contributions to the phenotypic correlations (**b**), for cerebellar volume across 28 regions and total cerebellum volume in the QTIM dataset. Lobules are presented in groups (anterior, superior posterior, inferior posterior, flocculonodular, vermis). Non-significant correlations/contributions are denoted **x** (significance adjusted for multiple comparisons), and negative estimates are shaded white). Twin models included corrections for effects of age, sex and ICV and specified only additive genetic (A) and unique environmental (E) sources of variance.


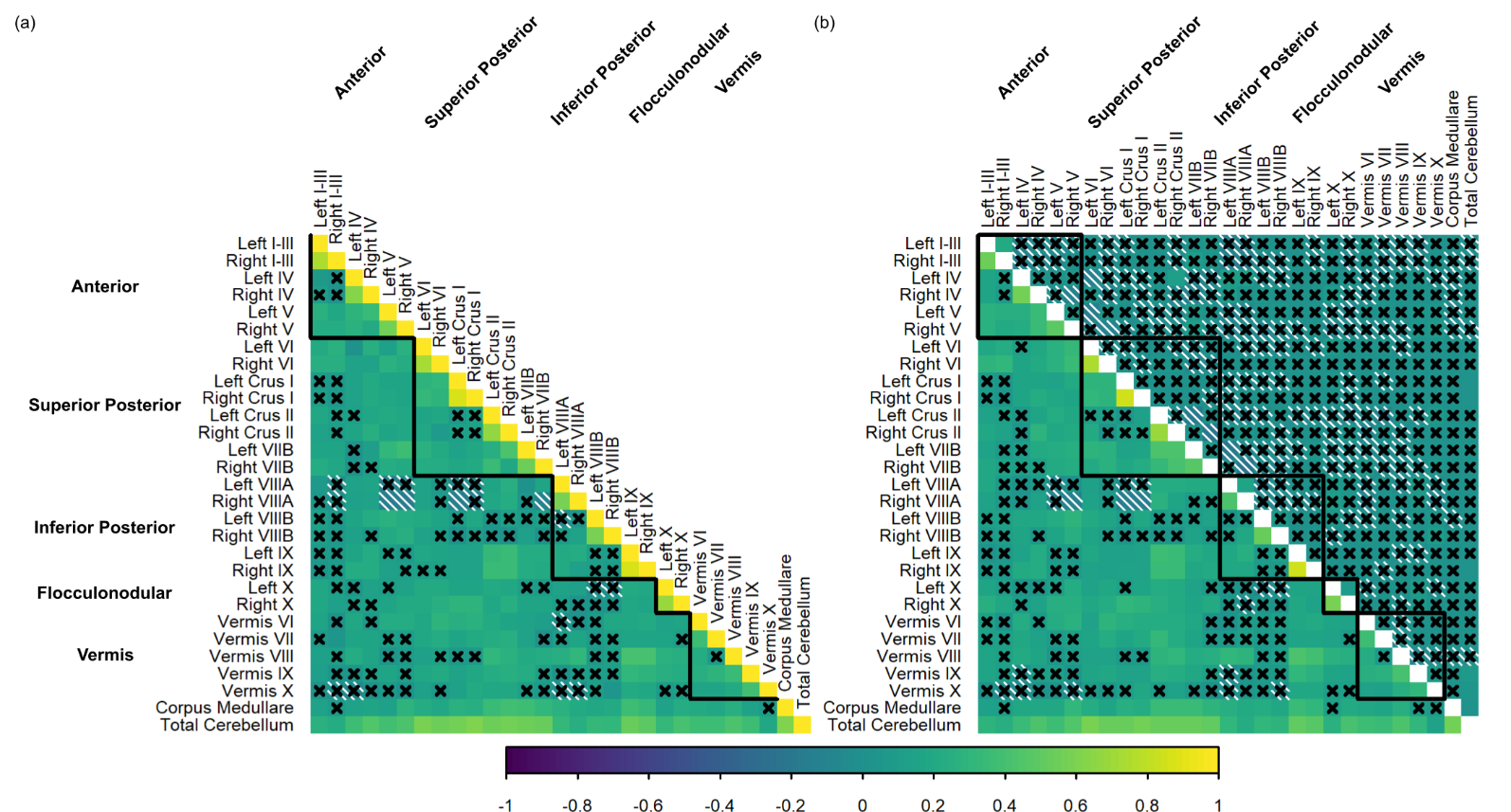


**FIGURE S3** Phenotypic correlations (**a**), with genetic (lower) and environmental (upper) contributions to the phenotypic correlations (**b**), for cerebellar volume across 28 regions and total cerebellum volume in the QTAB dataset. Lobules are presented in groups (anterior, superior posterior, inferior posterior, flocculonodular, vermis). Non-significant correlations/contributions are denoted **x** (significance adjusted for multiple comparisons), and negative estimates are shaded white. Twin models included corrections for effects of age, sex and ICV and specified only additive genetic (A) and unique environmental (E) sources of variance.

**
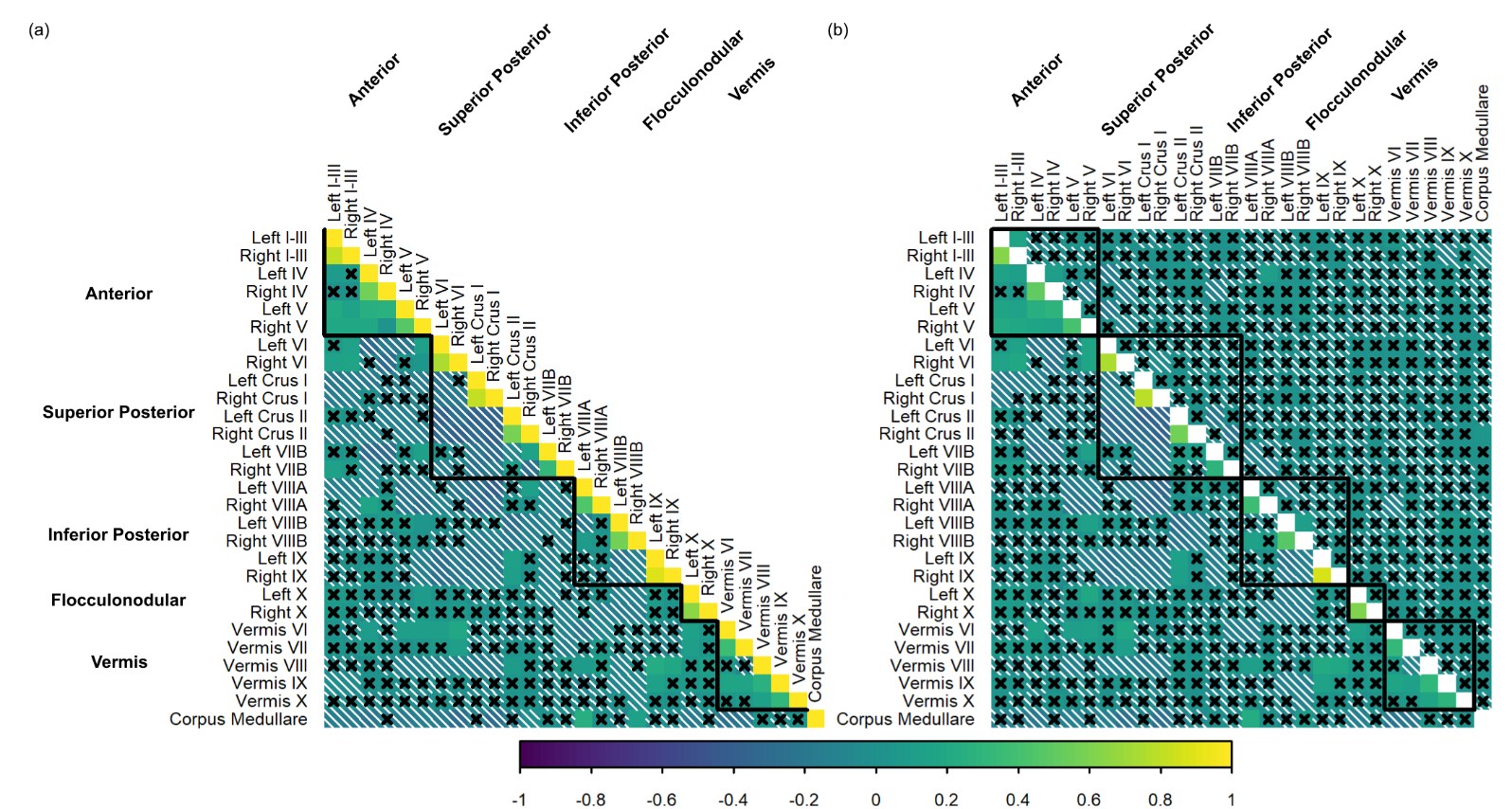
**

**FIGURE S4** Phenotypic correlations (a), with genetic (lower) and environmental (upper) contributions to the phenotypic correlations (b), for cerebellar volume across 28 regions in the pooled dataset (separate means, equated variance components). Lobules are presented in groups (anterior, superior posterior, inferior posterior, flocculonodular, vermis). Non-significant correlations/contributions are denoted **x**; negative correlations/contributions are shaded white). Twin models included corrections for effects of age, sex and total cerebellar volume (in place of ICV) and specified only additive genetic (A) and unique environmental (E) sources of variance.
